# Insights into the Management of Emerging Infections: Regulating Variant Creutzfeldt-Jakob Disease Transfusion Risk in the UK and the US

**DOI:** 10.1371/journal.pmed.0030342

**Published:** 2006-10-24

**Authors:** Maya L Ponte

**Affiliations:** Department of Anthropology, History, and Social Medicine, University of California San Francisco, San Francisco, California, United States of America; Sunnybrook and Women's College Health Sciences Centre, Canada

## Abstract

**Background:**

Variant Creutzfeldt-Jakob disease (vCJD) is a human prion disease caused by infection with the agent of bovine spongiform encephalopathy. After the recognition of vCJD in the UK in 1996, many nations implemented policies intended to reduce the hypothetical risk of transfusion transmission of vCJD. This was despite the fact that no cases of transfusion transmission had yet been identified. In December 2003, however, the first case of vCJD in a recipient of blood from a vCJD-infected donor was announced. The aim of this study is to ascertain and compare the factors that influenced the motivation for and the design of regulations to prevent transfusion transmission of vCJD in the UK and US prior to the recognition of this case.

**Methods and Findings:**

A document search was conducted to identify US and UK governmental policy statements and guidance, transcripts (or minutes when transcripts were not available) of scientific advisory committee meetings, research articles, and editorials published in medical and scientific journals on the topic of vCJD and blood transfusion transmission between March 1996 and December 2003. In addition, 40 interviews were conducted with individuals familiar with the decision-making process and/or the science involved. All documents and transcripts were coded and analyzed according to the methods and principles of grounded theory. Data showed that while resulting policies were based on the available science, social and historical factors played a major role in the motivation for and the design of regulations to protect against transfusion transmission of vCJD. First, recent experience with and collective guilt resulting from the transfusion-transmitted epidemics of HIV/AIDS in both countries served as a major, historically specific impetus for such policies. This history was brought to bear both by hemophilia activists and those charged with regulating blood products in the US and UK. Second, local specificities, such as the recall of blood products for possible vCJD contamination in the UK, contributed to a greater sense of urgency and a speedier implementation of regulations in that country. Third, while the results of scientific studies played a prominent role in the construction of regulations in both nations, this role was shaped by existing social and professional networks. In the UK, early focus on a European study implicating B-lymphocytes as the carrier of prion infectivity in blood led to the introduction of a policy that requires universal leukoreduction of blood components. In the US, early focus on an American study highlighting the ability of plasma to serve as a reservoir of prion infectivity led the FDA and its advisory panel to eschew similar measures.

**Conclusions:**

The results of this study yield three important theoretical insights that pertain to the global management of emerging infectious diseases. First, because the perception and management of disease may be shaped by previous experience with disease, especially catastrophic experience, there is always the possibility for over-management of some possible routes of transmission and relative neglect of others. Second, local specificities within a given nation may influence the temporality of decision making, which in turn may influence the choice of disease management policies. Third, a preference for science-based risk management among nations will not necessarily lead to homogeneous policies. This is because the exposure to and interpretation of scientific results depends on the existing social and professional networks within a given nation. Together, these theoretical insights provide a framework for analyzing and anticipating potential conflicts in the international management of emerging infectious diseases. In addition, this study illustrates the utility of qualitative methods in investigating research questions that are difficult to assess through quantitative means.

## Abstract 2

A qualitative study of US and UK governmental policy statements on the topic of vCJD and blood transfusion transmission identified factors responsible for differences in the policies adopted.

## Editors' Summary

**Background.:**

In 1996 in the UK, a new type of human prion disease was seen for the first time. This is now known as variant Creutzfeldt-Jakob disease (vCJD). Prion diseases are rare brain diseases passed from individual to individual (or between animals) by a particular type of wrongly folded protein, and they are fatal. It was suspected that vCJD had passed to humans from cattle, and that the agent causing vCJD was the same as that causing bovine spongiform encephalopathy (or “mad cow disease”). Shortly after vCJD was recognized, authorities in many countries became concerned about the possibility that it could be transmitted from one person to another through contaminated blood supplies used for transfusion in hospitals. Even though there wasn't any evidence of actual transmission of the disease through blood before December 2003, authorities in the UK, US, and elsewhere set up regulations designed to reduce the chance of that happening. At this early stage in the epidemic, there was little in the way of scientific information about the transmission properties of the disease. Both the UK and US, however, sought to make decisions in a scientific manner. They made use of evidence as it was being produced, often before it had been published. Despite this, the UK and US decided on very different changes to their respective regulations on blood donation. Both countries chose to prevent certain people (who they thought would be at greater risk of having vCJD) from donating blood. In the UK, however, the decision was made to remove white blood cells from donated blood to reduce the risk of transmitting vCJD, while the US decided that such a step was not merited by the evidence.

**Why Was This Study Done?:**

This researcher wanted to understand more clearly why the UK and US ended up with different policies: what role was played by science, and what role was played by non-scientific factors? She hoped that insights from this investigation would also be relevant to similar challenges in the future—for example, as many countries try to work out how to control the threat of avian flu.

**What Did the Researcher Do and Find?:**

The researcher searched for all relevant official government documents from the US and UK, as well as scientific papers, published between the time vCJD was first identified (March 1996) and the first instance of vCJD carried through blood (December 2003). She also interviewed people who knew about vCJD management in the US and UK—for example, members of government agencies and the relevant advisory committees. From the documents and interviews, the researcher picked out and grouped shared ideas. Although these documents and interviews suggested that policy making was rooted in scientific evidence, many non-scientific factors were also important. The researcher found substantial uncertainty in the scientific evidence available at the time. The document search and interviews showed that policy makers felt guilty about a previous experience in which people had become infected with HIV/AIDS through contaminated blood and were concerned about repeating this experience. Finally, in the UK, the possibility of blood contamination was seen as a much more urgent problem than in the US, because BSE and vCJD were found there first and there were far more cases. This meant that when the UK made its decision about whether to remove white blood cells from donated blood, there was less scientific evidence available. In fact, the main study that was relied on at the time would later be questioned.

**What Do These Findings Mean?:**

These findings show that for this particular case, science was not the only factor affecting government policies. Historical and social factors such as previous experience, sense of urgency, public pressure, and the relative importance of different scientific networks were also very important. The study predicts that in the future, infectious disease–related policy decisions are unlikely to be the same across different countries because the interpretation of scientific evidence depends, to a large extent, on social factors.

**Additional Information.:**

Please access these Web sites via the online version of this summary at http://dx.doi.org/10.1371/journal.pmed.0030342.

- National Creutzfeldt-Jakob Disease Surveillance Unit, Edinburgh, UK
- US Centers for Disease Control and Prevention pages about prion diseases
- World Health Organization variant Creutzfeldt-Jakob disease fact sheet
- US National Institute of Neurological Disorders and Stroke information about prion diseases

## Introduction

Avian influenza is the latest in a long line of zoonoses, or diseases transmissible from animal to human, to raise the specter of an impending epidemic of human-to-human transmission [1]. Solidarity amongst nations in their approach to this threat is deemed to be one key to a successful response [2]. The other is careful use of scientific data to sculpt a rational strategy [3]. The two are felt to go hand in hand, as it is often inferred that a reliance on science will lead to appropriate and harmonious policies amongst nations.

As efforts are already being made in many countries to protect against an H5N1 epidemic, now is a good time to reflect on whether or not the use of science necessarily leads to agreement amongst nations and what factors, other than science, play a role in shaping disease management policy. In this vein, a case study of variant Creutzfeldt-Jakob disease (vCJD) is illustrative. vCJD is a prion disease that originated from the transfer of bovine spongiform encephalopathy (BSE), or “mad cow disease,” from cattle to humans [4]. Almost immediately after the disease was first reported in 1996, concerns were raised about the possibility of transmission of the disease between humans through blood transfusion [5]. The risk was purely hypothetical in nature, as there was no evidence of transfusion transmission having taken place,. The first probable case of transfusion-transmitted vCJD would not be identified until late 2003 [6], the second in 2004 [7]. And yet, many nations implemented regulations aimed at reducing the risk of such transmission while the risk was still hypothetical in nature. This study inquires into the factors that influenced the design of regulations to reduce the risk of transfusion transmission of vCJD in the UK and the US before December 2003.

Prion diseases are fatal human and animal neurological disorders defined by their transmissibility and characteristic neuropathology [8]. Scrapie, a prion disease of sheep, was first described more than 200 years ago [9]. In the 1950s, scrapie spread from Europe to many other parts of the world, including the US. The most common human form of prion disease is sporadic Creutzfeldt-Jakob disease (sCJD). The incidence of sCJD is approximately one out of 1 million people per year, in each country around the world [10]. Certain populations are also plagued by familial forms of prion disease, including Gerstmann-Straussler syndrome [11] and familial CJD [12].

CJD was first perceived as an infectious threat to developed nations when contaminated human growth hormone and dura mater grafts transmitted the disease to more than 250 individuals in the 1980s and 1990s [13]. However, it was the emergence of the BSE epidemic among British cattle in 1986, and the transmission of BSE to humans in the form of vCJD [14], that provided the major impetus to develop coherent disease management policies in many nations. So far, more than 177 individuals have succumbed to vCJD, all but 21 of them UK residents [15]. Only one US resident, who spent much of her childhood in the UK, has been diagnosed with the disease [15].

The sense of urgency that accompanied vCJD was felt most strongly in the UK, where the number of cattle diagnosed with BSE had topped 170,000 by 1997 [16]. Some biostatisticians estimated that this was just the tip of the iceberg, representing only a fraction of the actual number of cases [17]. The US did not detect BSE in its cattle herds until 2003 [18]. And yet, vCJD became a concern in the US due to the number of US military troops stationed in the UK during the peak of the BSE epidemic and the volume of travel between the two countries [19].

Thus, US and UK regulatory agencies convened meetings of their scientific advisory committees to evaluate the literature, consider what was known about the disease, and debate an effective means of preventing further spread. Both nations quickly identified potential transfusion transmission of vCJD as a risk that required regulation. In assessing this risk, the cost of limiting the blood supply was weighed against the potential harm of a blood-borne vCJD epidemic. Both nations, in parallel fashion, ultimately chose to restrict certain portions of the donor pool. The US and UK, however, developed strikingly different positions regarding the removal of white blood cells from blood components as a strategy for reducing vCJD transmission risk. The process of removing white blood cells from blood components is referred to by a variety of terms, including leucocyte depletion, leucodepletion, leukocyte reduction, and leukoreduction. The UK determined that leukoreduction would likely reduce the transmission risk, leading to a policy of universal leukoreduction. In the US, on the other hand, it was felt that leukoreduction would have a negligible effect on transmission risk and therefore did not merit implementation for this purpose. Despite this difference, both US and UK regulatory agencies presented their strategies as grounded in scientific evidence.

The questions that motivated this investigation were: (1) How did transfusion transmission of vCJD become a prominent concern of regulatory agencies in the US and UK? (2) What role did science play in deliberations concerning appropriate regulatory policy in each country? (3) What non-scientific factors influenced the regulatory process in each country? (4) And finally, if both the US and UK carried out their deliberations in a science-based manner, how did their processes result in policies that are parallel with respect to sourcing of plasma but incongruous with respect to leukoreduction of blood components?

The goal of qualitative research is the development of concepts that aid in the understanding of social phenomena [20]. As such, the goal of this study is not to “prove” or “verify” a testable hypothesis, but to provide a set of theoretical insights that illuminate the processes involved in the management of emerging infectious diseases.

## Methods

### Initial Data Collection

The data for this paper are part of a larger study on prion disease in the US and UK that was conducted between October 2001 and July 2005. For this subsection of the study, a background document search was conducted to identify US and UK governmental announcements, policy statements, documentation of governmental scientific advisory committee meetings (including those of the US Food and Drug Administration Transmissible Spongiform Encephalopathy Advisory Committee, the US Department of Health and Human Services Blood Safety and Availability Committee, the UK Spongiform Encephalopathy Advisory Committee, the UK Advisory Committee on the Microbiological Safety of Blood and Tissues for Transplantation, and the UK Committee on the Safety of Medicines), research articles, and editorials published on the topic of vCJD and blood transfusion transmission between the identification of vCJD (March 1996) and the identification of the first likely case of transfusion-transmitted vCJD (December 2003). When transcripts of advisory committee meetings were available, transcripts were always analyzed in preference to minutes or summaries. When transcripts were not available, minutes, summaries, and other supporting documentation were analyzed. All document searches were restricted to the period from March 1, 1996, to December 31, 2003, unless otherwise specified.

UK governmental documents pertaining to the regulation of vCJD with respect to the blood supply were identified through online sources (Figure 1). The official Spongiform Encephalopathy Advisory Committee Web site (http://www.seac.gov.uk/papers/papers.htm) maintains archives of SEAC meetings, from October 1997 onward. A search of the text of minutes or summaries (when minutes were not available) of the meetings that occurred between October 1997 and December 2003 for the term “blood” yielded 20 results. Six of the identified meetings were excluded from analysis because the discussion did not pertain to human blood products or blood transfusion (for example, the spreading of bovine blood on fields as fertilizer). All documentation available on the Web site from the remaining 14 meetings was included in the analysis. A manual search of the scanned documents dating from March 1, 1996, or later contained within the BSE Inquiry Web site Yearbook section (http://www.bseinquiry.gov.uk/evidence/yb/index.htm), comprised of primary evidence gathered during the inquiry, led to the identification of nine additional documents in which blood transfusion was discussed in relation to vCJD. These documents pertained to meetings of SEAC and other committees, including three meetings of COHASE (Committee on the Human Aspects of Spongiform Encephalopathies).

**Figure 1:**
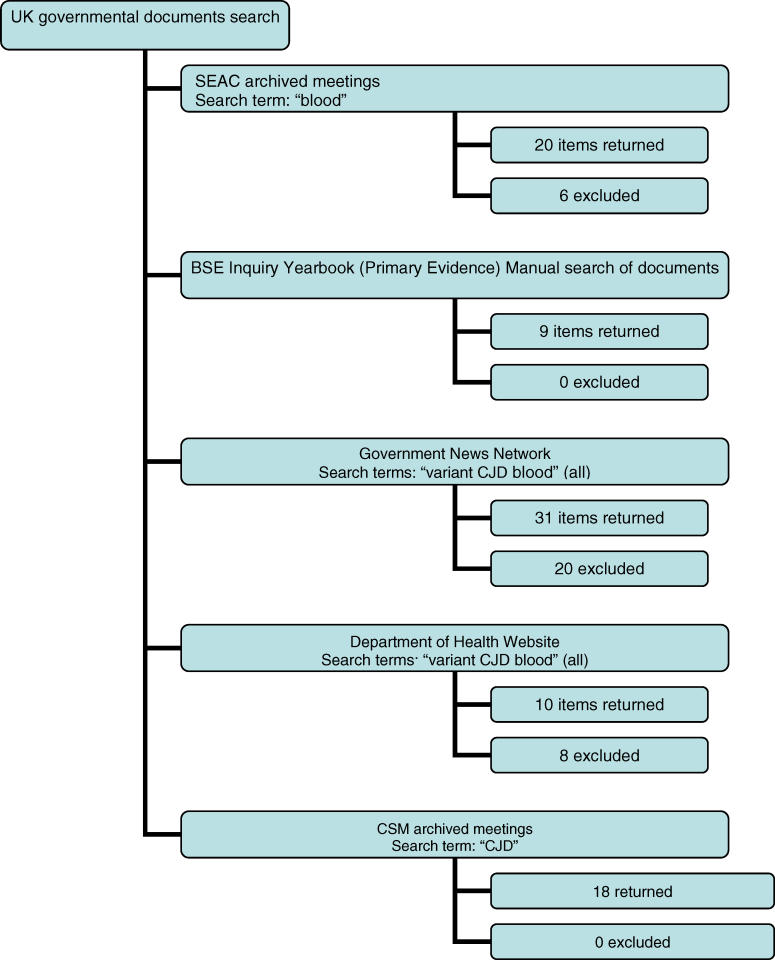
UK Government Documents Searches conducted with results returned and excluded at each step.

A search of the Government News Network site (www.gnn.gov.uk), a repository for press releases and other public documents of UK governmental departments, using the terms “variant CJD blood” and selecting “all words” led to the identification of 31 documents relating to the topic between March 1996 and December 2003. After duplicates were excluded, 12 items remained. One was a summary of a SEAC meeting that had already been identified in a previous search, leaving 11 unique documents. Two of the eleven documents identified were summaries of meetings of the Advisory Committee on the Microbiological Safety of Blood and Tissues for Transplantation (MSBT). An advanced search of the Department of Health Web site (www.dh.gov.uk/AdvancedSearch/fs/en) of all documents from March 1996 to December 2003 for all of the words “variant CJD blood” returned ten documents, eight of which had already been identified in the previous search and two of which were newly identified and included in the analysis.

The Medicines and Healthcare Products Regulatory Agency (MHRA) of the UK Department of Health maintains archives of meeting minutes of the Committee on Safety of Medicines (CSM) from January 1998 onward at its Web site (http://www.mhra.gov.uk). A search of the minutes of all meetings through December 2003 for the term “CJD” led to the identification of 18 documents. Minutes were included in the analysis if the documented discussion pertained to vCJD. All documents met this criterion and were included in the analysis.

US governmental documents pertaining to the regulation of the vCJD transfusion risk were identified through several online sources (Figure 2). A search of the text of the agendas of archived TSEAC meetings on the FDA dockets Web site (http://www.fda.gov/ohrms/dockets/) for the term “blood” led to the identification of 11 meetings. Documents were included in the analysis if they dealt with the topic of transfusion transmission of vCJD. All of the meetings identified met this criterion. Full transcripts were available for analysis of all 11 meetings. Also at the FDA dockets Web site, the agendas and transcripts of archived Blood Products Advisory Committee (BPAC) meetings were searched for the terms/phrases “vcjd”, “variant cjd”, or “variant creutzfeldt”, leading to the identification of 16 meetings that included at least one instance of one of these terms. One was a joint meeting with TSEAC and was thus a duplicate of a document already identified. Three meeting transcripts were excluded from analysis because the mention of vCJD was incidental to the discussion of another topic. In sum, the BPAC archives search led to the identification of 12 additional meeting transcripts that were included in the analysis. While the dockets Web site is inclusive of meetings from January 1997 to the present, searches were restricted to those meetings occurring before December 2003.

**Figure 2:**
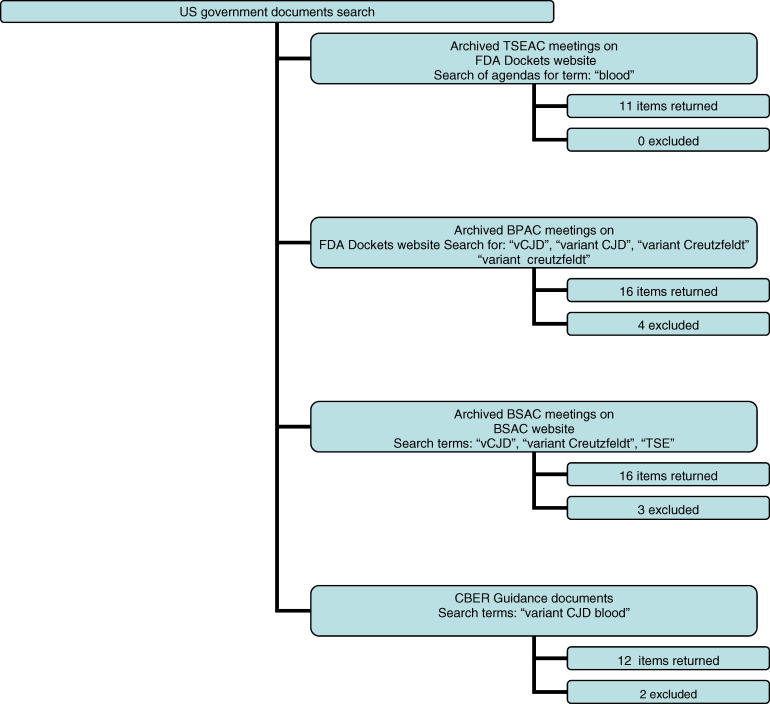
US Government Documents Searches conducted with results returned and excluded at each step.

A search of the text of transcripts of archived Blood Safety and Availability Committee (BSAC) meetings on the BSAC Web site (http://www.hhs.gov/bloodsafety/) for the phrase “variant CJD” led to the identification of 16 meetings at which vCJD was mentioned. Three of the meeting transcripts were excluded from analysis because the mention of vCJD was brief and incidental to another topic of discussion. The remaining 13 meeting transcripts were analyzed as data. A search of guidance documents published by the Center for Biologics Evaluation and Review (CBER) of the FDA (www.fda.gov/cber/guidelines.htm) using the terms “variant CJD blood” led to the identification of 12 documents. After duplicates were excluded, ten items remained and were included in the analysis.

Publications in scientific and medical journals related to blood transfusion and vCJD, CJD, or leukoreduction were identified by searching the National Library of Medicine's MEDLINE database using PubMed (Figure 3). A PubMed search for the text words “CJD” or “Creutzfeldt-Jakob” and “blood” identified 162 items that were published before 31 December, 2003. Documents were excluded from analysis if they were in a language other than English or if they were of a topic that was considered to be unrelated to CJD or vCJD and blood infectivity or blood transfusion risk. These criteria led to the exclusion of 45 items from analysis. A PubMed search for the phrases “universal leucocyte depletion” or “universal leukocyte depletion” or “universal leukoreduction” or “universal leukocyte reduction” yielded 38 results that were published before 31 December, 2003. Documents were excluded from analysis if they had already been identified in a previous search or if they were of a topic involving an aspect of universal leukoreduction that was considered to be irrelevant to the analysis. These criteria led to the exclusion of eight documents. In sum, a total of 147 unique scientific articles, scientific reviews, policy reviews, editorials, and commentaries were included in the analysis.

**Figure 3:**
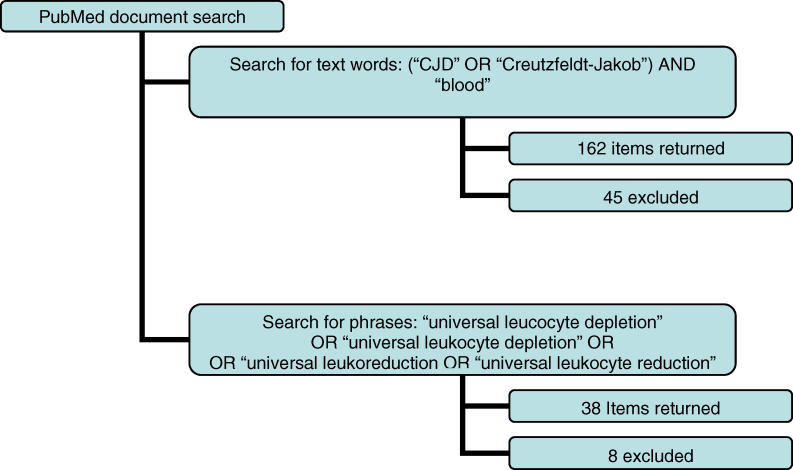
Scientific Publications, Editorials, and Reviews Searches conducted with results returned and excluded at each step.

### Selection of Participants

Forty interviews were conducted with individuals familiar with the decision-making process in the US or the UK. The goal was to target the individuals who were most knowledgeable about the way decisions were made regarding the management of vCJD in each country. Situational, rather than demographic, representativeness was desirable. Because some decisions and recommendations were made in the course of advisory committee meetings and others within the agencies, it was decided that a situationally representative sample would include members of US and UK advisory committees as well as relevant government agencies. For purposes of simplification, only the advisory committee that was most influential in determining the course of vCJD management with respect to the blood supply, as judged by document review, was targeted in each country. In the US, this was the Transmissible Spongiform Encephalopathy Advisory Committee (TSEAC), and in the UK this was the Spongiform Encephalopathy Advisory Committee (SEAC).

In the US, all government employees who participated in this portion of the study were employed by the Department of Health and Human Services (DHHS). In the UK, the government employees who were recruited for interview came from the Department of Health as well as the National Blood Service for England and Wales and the Scottish National Blood Transfusion Service. Recruitment was conducted in a stepwise fashion with the goal of recruiting approximately equal numbers of participants from the US and UK, with roughly twice as many advisory committee members as government employees. First, 20 individuals who were members of TSEAC or SEAC or who were government agency employees involved in the management of vCJD were invited to participate in the study. These individuals were selected based on a number of considerations, including the time period during which they served on the government advisory committee or in the government, their educational background, their current position, and information pertaining to their contributions that was derived from background documents. Specifically, individuals were recruited to represent different backgrounds and to cover critical periods of time in the decision-making process. Individuals were also recruited based on the importance of the contributions they made to the decision-making process as determined by the document review. During interviews, other individuals important to the decision-making process were identified and further contacts were made. While most of these additional contacts were also members of the advisory committees or government employees, four were scientists who were knowledgeable about the decision-making process or the science involved, though they did not officially serve on advisory committees. In this way, recruitment and data collection was an iterative process.

Of the 14 current or former members of the UK Spongiform Encephalopathy Advisory Committee who were invited to participate in the study, 12 were ultimately interviewed. Of the members of SEAC who participated, nine were university professors. Six held advanced scientific degrees, three held medical degrees, and one held a non-medical, non-scientific degree. Of the three who were not university professors, two held degrees in veterinary medicine, one with experience in private practice and the other with experience in research. Of the two who declined to participate in the study, one was a university professor with an advanced scientific degree and the other was a university professor with a degree in medicine.

Of the 13 current or former members of the US Transmissible Spongiform Encephalopathy Advisory Committees contacted, 12 agreed to participate and were included in the study. Of the 12 who ultimately participated, six were professors at research universities. Of these, three held a medical degree and three held advanced scientific degrees. Five of the participants were researchers at government agencies, one with a degree in medicine, two with advanced scientific degrees, and two with veterinary degrees. One was a representative from a consumer rights organization with a degree in medicine. The individual who declined to participate was a university professor with a medical degree.

All six of the current or former employees of UK government agencies (Department of Health, National Blood Service for England and Wales, and the Scottish National Blood Transfusion Service) who were asked to participate agreed to be interviewed. Three held medical degrees, two held advanced scientific degrees, and one held a non-medical, non-scientific degree. Four had more than 20 years' experience in UK government agencies, and two had fewer than 20 years' experience.

Of the seven current or former employees of US government agencies (Department of Health and Human Services) that were invited to participate, six agreed to be interviewed. Of the six who participated, four held degrees in medicine and two held advanced scientific degrees. Half had more than 20 years' experience in government, and half had less than 20 years' experience in government. The one who did not participate had a medical degree and more than 20 years' experience in government.

Four individuals with advanced scientific degrees who were not governmental employees or scientific advisers were identified by members of SEAC or TSEAC as knowledgeable about the decision-making process and were also invited to participate. All four ultimately participated. Three were professors in the UK, and one was a professor in the US.

The final tally of participants included six current or former employees of UK government agencies (Department of Health, National Blood Service for England and Wales, and the Scottish National Blood Transfusion Service) and six current or former employees of US government agencies (Department of Health and Human Services), 12 current or former members of the UK Spongiform Encephalopathy Advisory Committee, 12 current or former members of the US Transmissible Spongiform Encephalopathy Advisory Committee, and four scientists who belonged to neither group.

### Interviews

Most interviews were conducted in person at a site that was convenient for the interviewee. A small number of interviews were conducted on the phone. In a few cases, more than one interview was required to cover all the topics. The interviews were conducted in an open-ended, semi-structured manner, a technique common in ethnographic research [21]. While there was no specific order to the questions that were asked, it was ensured that the interview would cover the four topics listed at the end of the introduction. Interviews were recorded and transcribed verbatim.

### Analysis

All data, including interview transcripts, transcripts or minutes of advisory committee meetings, agency announcements, and scientific literature, were analyzed using the general principles of grounded theory [22,23]. Grounded theory is a qualitative methodology that allows for the study of topics in medicine and policy that are difficult to investigate through deductive, quantitative mechanisms. For example, researchers have used variations of grounded theory to ask why people delay seeking care after a heart attack [24] and how physicians and nurses decide whether to report a medical error [25].

Grounded theory focuses on uncovering the social processes and conditions that lie behind the phenomena in question and following their consequences and effects. It offers a systematic process through which concepts can be developed inductively. Rather than beginning with a hypothesis, the researcher begins with a set of questions. Codes are then generated through the labeling of concepts, and the concepts are grouped into categories. Conceptual categories are then refined in terms of their properties and variations through the analysis of further data. Connections between categories are labeled through a process referred to as axial coding, eventually forming the basis for constructing major categories.

Specifically, data analysis proceeded as follows. First, data were stratified by country (US versus UK), source (regulatory, scientific advisory committee, etc.), and regulation strategy under discussion (leukoreduction, deferral of donors, etc.). Descriptive codes were then developed based on the type of information cited (scientific, experiential, etc.), the origin of the material, and the role it played in the consideration of various risks and policy interventions. Through a comparison of the resulting codes, numbering more than 100, 22 major categories were derived. Categories developed in the analysis of data from documents were compared to those developed through the analysis of interview data, allowing for cross-validation between the categories derived from different types of data. As a check on the validity of the author's interpretations, one respondent from each of the five groups interviewed (members of SEAC, members of TSEAC, UK government employees, US government employees, and other) was consulted regarding the appropriateness of the categories that pertained to his or her participant group. From the resulting final categories, a series of theoretical insights were derived by the author, three of which will be described in this paper.

## Results

### Existing Scientific Evidence and Uncertainty

Government agency employees and scientific advisors from both the US and UK were quick to point to a scientific basis for the concern about transfusion transmission of vCJD. They also pointed to the uncertainty, however, that accompanied the interpretation of the evidence. Prior to the appearance of vCJD, several studies had detected infectivity in the blood of rodents with prion disease [26–28]. These data, along with the growing number of human growth hormone and dura mater transmitted cases of sCJD [29,30], led to the development of concern that sCJD could be transmissible through blood transfusion [28,31]. Several countries, including the US, instituted policies aimed at preventing the transmission of sCJD through blood [32]. Concern about this route of transmission, however, was mitigated by epidemiological studies that reported no association between blood transfusion and sCJD [33–35] and surveillance studies that failed to detect cases of sCJD in frequent users of blood products, such as people with hemophilia [36]. Despite the reassuring evidence on sCJD, vCJD was viewed as an entirely new disease with potentially concerning properties. Unlike sCJD, infectivity was detected in the lymphoreticular tissue, such as tonsil, appendix, and lymph nodes, of patients with vCJD [37,38]. Because this pattern was similar to that found in rodents with prion disease, it led to speculation that vCJD, like rodent-adapted prion disease, could be transmissible through blood. There was no direct evidence, however, to implicate the blood of individuals with vCJD as infectious. The concern was therefore considered hypothetical in nature.

The weight granted to this hypothetical concern derived in part from the uncertainty surrounding the magnitude of the vCJD epidemic. With no ante-mortem diagnostic test capable of detecting prion disease in humans, it was impossible to assess the number of individuals infected. The unknown, yet potentially lengthy, incubation period made this number difficult to estimate. As a result, early predictions of the size of the vCJD epidemic ranged from as few as 75 people to as many as 13.7 million people [39,40].

### Donor Selection Criteria: The Influence of the HIV Experience in the US

The existing prion disease science and the uncertainty surrounding the vCJD epidemic, however, were not the only factors that led to a focus on transfusion as a potential transmission risk of vCJD. All but one of the US government agency employees interviewed stated that if it had not been for past experience with HIV, and to a smaller extent hepatitis C, it is unlikely that the issue of transfusion transmission of vCJD would have garnered so much attention. The single employee who did not support this position had not participated directly in the management of HIV at the agency. According to the remainder of the government employees interviewed, the history of HIV/AIDS is something that had come to haunt US regulatory agencies, especially the Food and Drug Administration (FDA). This stems from a decision made by the FDA in 1983 not to call for removal from the market of plasma products from donors with AIDS [41]. After a widespread epidemic of transfusion transmissions ensued, the FDA was called to task for this decision by hemophilia activists, by the US Congress, and by various news organizations. One US government employee suggested that when it came to regulating blood, the fallout from HIV/AIDS continues to affect decision-making at the agency “every minute of every day.”

Along these lines, half of the US government agency employees also mentioned the influence of the Institute of Medicine (IOM) report on HIV transmission through blood and blood products. This report, published in 1995, a year before vCJD was discovered, recommended that a precautionary stance be taken with regard to potential future threats to the blood supply [41]. Uncertainty, the report claimed, should not be an excuse for inaction, especially when it came to the efforts by the FDA to regulate blood and blood products.

This history loomed large as the DHHS brought questions about the potential transmissibility of vCJD through blood to the Blood Products Advisory Committee (BPAC), the Transmissible Spongiform Encephalopathy Advisory Committee (TSEAC), and the Blood Safety and Availability Committee (BSAC). In addition, there was a heightened state of concern and political activism within the hemophilia community due to recent experience with HIV/AIDS. Tens of thousands of people with hemophilia had contracted the disease through contaminated plasma products, and the community was wary of another threat to transfusion safety.

According to all but one of the US government agency employees interviewed, hemophilia activists played a major role in ensuring that the concern about vCJD transfusion transmission became a focus at the agency. They did this by writing letters, arranging meetings with staff, and attending and speaking at advisory committee meetings. This contention is supported by government documents [19,42,43] as well. The single US government employee who did not feel that hemophilia activists played a major role in ensuring that the concern about vCJD transfusion transmission became a focus at the agency was the same employee who felt that the HIV/AIDS experience had not influenced the management of vCJD.

Three-quarters of the members of TSEAC who were interviewed expressed their belief that the recent experience with transfusion transmission of HIV/AIDS played a significant role in creating concern about the hypothetical risk of transfusion transmission of vCJD. They felt that the impassioned speeches of hemophilia activists at advisory committee meetings influenced the direction of the discussion and the recommendations made. In addition, a few members of TSEAC were influenced by their personal experience in advising on blood policy during the HIV epidemic.

The first strategy that was considered by the FDA and its TSE advisory committee for reducing the exposure to vCJD through the blood supply was to disqualify donors who had spent time in the UK and other countries in which BSE had been reported [19]. This strategy, however, conflicted with the goal of maintaining an adequate blood supply. As a result, discussion at advisory committee meetings centered on balancing the potential benefit achieved through donor exclusion criteria with the potential risk of reducing the blood supply [19,43,44].

Blood collection organizations presented the advisory committees with information and projections regarding the effects of various donor selection criteria on the blood supply. Based on a survey, the American Red Cross (ARC) estimated a loss of 11.7% in blood donations if everyone who visited the UK between 1984 and 1990 was excluded [19]. Representatives of the ARC expressed their concern that recruitment efforts would not be able to make up for such a loss. The American Association of Blood Banks (AABB), estimating a loss of 1.4 million to 2 million units each year, projected devastating problems for the blood supply [19].

Members of the hemophilia community attended advisory committee meetings and demanded that maintaining an adequate supply should not overshadow safety concerns [19,42,43,45]. They pointed out that such concerns had prevented the appropriate management of HIV with respect to the blood supply. An example of this type of speech is exhibited by Jan Hamilton of the Hemophilia Federation at a 1998 BSAC meeting:

Does anyone hear an echo? An echo which eerily reminds us of the early 1980s? Are we once again being led down a path which will attack and eradicate several thousand more persons with hemophilia? We are constantly being put in the position of having to take the risks and our community is woefully tired of being the canaries in the coal mine … [42]

This line of argument was subsequently taken up by members of the advisory committees. For example, Barbara Harrell, a consumer representative on TSEAC, remarked at a 1998 meeting that: “the reluctance to reduce the repeat donor pool to reduce the theoretical risk of HIV allowed that disease to become epidemic in the United States” [19].

In order to balance these two competing concerns, members of TSEAC recommended restricting donor deferral to individuals who had spent a significant amount of time in the UK. Because the majority of US tourists visit the UK for only a short amount of time, it was suggested that such a measure would limit losses to the blood supply while excluding the riskiest donors. To aid in their selection of the appropriate criteria, committee members asked the blood-banking community to conduct another survey that would specify the amount of time blood donors had spent in the UK [19].

The results from this survey led to the introduction of a tiered system of deferrals based on the estimated loss of donors at each level. In November 1999, the FDA instituted deferrals for anyone who had spent more than six months in the UK between 1980 and 1996 [46]. In May 2002 they decreased the allowable amount of time in the UK to three months and added a deferral for members of the US military who had been stationed in certain other parts of Europe for more than six months [46]. In October 2002, a deferral was instituted for all donors who had spent a total of more than five years in Europe from 1980 to the present [46]. At each of these steps, the number of donors likely to be lost was weighed against the potential risk reduction [43,44].

### Donor Selection Criteria: The Influence of the HIV Experience in the UK

In the UK at that time, unlike the US, governmental advisory committee meetings were closed to the public. While hemophilia activists were unable to attend these meetings, they found other avenues through which to make their voices heard. An example of this is an open letter published in *The Lancet* by the United Kingdom Haemophilia Centre Directors' Organization (UKHCDO), demanding access for people with hemophilia to plasma products imported from countries thought to be free of BSE [47]. In their letter, the UKHCDO explicitly drew on past experience with AIDS in calling for such regulations.

Following the UKHCDO's letter, *Nature* reported that “foot-dragging by the British government is exposing hemophiliacs to an avoidable risk of infection with the new variant of Creutzfeldt-Jakob disease” [5]. Although such a measure had not been recommended by SEAC, UK Health Minister Frank Dobson announced in February 1998 that UK plasma fractionation centers would begin importing plasma from BSE-free countries for the production of plasma products. In his reasoning, Dobson explicitly noted that while the risk of vCJD transmission “is hypothetical, nevertheless the fear of it is very real to this group which has previously been affected by both HIV and Hepatitis C transmitted from Factor VIII” [48].

As in the US, the selection of appropriate donor restriction criteria in the UK was influenced by competing interest in maintaining an adequate blood supply. Significantly, the donor selection restrictions in the UK were applied to only plasma products and not labile components such as packed red blood cells. Due to their limited shelf life, there was concern in the UK about maintaining an adequate supply of labile components from overseas sources.

In interviews, all UK governmental employees said that the experience with HIV/AIDS played a crucial role in shaping concern about vCJD transmission through blood and plasma products, especially through concerns raised by the hemophilia community. Evidence for this is found in government documents as well [49]. Half of the government employees mentioned the threat of recalls of plasma products for possible vCJD contamination, as occurred in October and November of 1997 in the UK [50], in contributing to the decision to import plasma. Government employees pointed out that recalls are expensive to carry out and often much of the product has already been consumed by the time of the recall, leading to fear of exposure on the part of consumers. Just prior to the UK's decision to import plasma, the Committee for Proprietary Medicinal Products, Europe's pharmaceutical regulator, banned pharmaceuticals containing UK-derived albumin, a measure designed to avoid such recalls [51]. While mentioned in only one interview, this likely played a role in the UK's decision-making process, as evidenced by documentation of meetings held by the Committee on the Safety of Medicines [52–54].

While two-thirds of the members of SEAC felt that the background of HIV/AIDS contributed to the focus on blood as an avenue for transmission of vCJD, none of the members of SEAC felt that hemophilia activists had a direct impact on the flow of the discussion or the decision making by committee members. Such an outcome is not surprising considering that hemophilia activists were not present at SEAC meetings. This observation is also consistent with the fact that SEAC did not recommend the importation of plasma at their 24 October 1997 meeting.

### Consideration of Leukoreduction as a Risk Management Strategy in the UK

At their 24 October 1997 meeting, SEAC recommended “that the Government should consider a precautionary policy of extending the use of leucodepleted blood and blood products as far as is practicable” [55]. Leucodepletion, often referred to as leukoreduction in the US, is defined as the removal of white blood cells by filtration or other approved methods so that less than a specified number of lymphocytes remain in the final product [56]. The members of SEAC interviewed who were serving on the committee at the time agreed that one particular scientific study became the focus of discussion and motivated their decision to recommend leukoreduction. This study, conducted by Adriano Aguzzi's lab at the University of Zurich, had not yet been published. The committee was made aware of the study prior to publication through the existence of social and professional connections between Aguzzi's lab and scientists and regulators in the UK.

Aguzzi's experiment employed a variety of transgenic mouse models to clarify which elements of the immune system were required for prion infection [57]. Some of the mice were missing genes necessary for the production of B and T cells, some for the production of B cells only, and others for the production of T cells only. Aguzzi's group reported that when exposed, the mice deficient in functional T cells developed the disease normally while those deficient in B cells were protected. They concluded that “B cells may be the physical carriers of prions” in the blood [57].

While Aguzzi's results did not prove that B cells carry prions, scientific advisors and regulators were intrigued by this possibility. It implied that merely removing B cells from blood products could reduce the likelihood of transmitting infection. On this basis, SEAC suggested that “it is logical to seek to minimize any risk from blood or blood products by reducing the number of lymphocytes present” [55].

Members of SEAC viewed Aguzzi's results as consistent with the results of prior experiments on blood that had detected infectivity through assay of the buffy coat, a component of blood in which lymphocytes are concentrated. The buffy coat, in fact, had been the sole component of blood to be assayed in many of these studies [26,27]. This was due to the notion that prion infectivity should be cell-associated. In other words, most researchers had not looked for infectivity in plasma or other blood components.

All of the UK government employees identified the results of this experiment as having a significant influence on the subsequent decision of the UK Department of Health to implement SEAC's recommendation. More than half indicated that if the political environment at the time had not necessitated immediate action on the issue, the results of a single experiment would never have been relied on to such an extent. Pressure from the public was building in light of the identification of several vCJD victims who had previously donated blood. In late October and early November of 1997, the UK government issued recalls of blood products for possible vCJD contamination [50]. In both cases, most of the implicated blood products had already been consumed. The recalls were highly publicized, and the resulting public concern raised the stakes for risk reduction efforts. Two days after the second recall announcement, Health Minister Frank Dobson issued a press release stating that the government had accepted SEAC's advice related to vCJD and had instructed the NBS “to start work towards the possible extension of leucodepletion of blood” [58].

In July 1998, after the NBS had developed the appropriate infrastructure for managing universal leukoreduction, the policy was officially implemented [59]. In the interim between the government's acceptance of SEAC's recommendation and its implementation, Lord Phillips' inquiry into the management of BSE had begun [60]. New revelations about the mishandling of the BSE crisis appeared in the papers daily, increasing the pressure on the government to do something. In announcing the implementation, Frank Dobson said: “We will do whatever we are advised to reduce the theoretical risk to the blood supply of the transmission of nvCJD” [59].

Even at the time, a reduction in vCJD transfusion transmission risk was not viewed as the only potential benefit of leukoreduction. As the Deputy Chief Medical Officer, Jeremy Metters, noted: “There are a variety of benefits for patients attributed to the use of leucodepleted blood. It avoids the risk of fever in patients who require repeated transfusions, reduces the risk of graft rejection in patients requiring bone marrow transplants, and prevents infections in babies younger than a year” [59]. It was estimated by the NBS, however, that such identifiable clinical benefits could not justify the cost of implementing universal leukoreduction [61]. Instead, Dobson and Metters emphasized that “SEAC's expert advice is that leucodepletion would be a sensible and practical precautionary measure to take against the theoretical risk from [vCJD] because if infectivity were to be present in blood, it would most likely be in the white cells” [59].

While a risk assessment of vCJD transfusion transmission was commissioned on the advice of SEAC, the majority of the government employees and members of SEAC who were interviewed felt that it had little impact on the actual decision-making process. Support for this conclusion is found in documentation indicating that both the policy to import plasma and the policy to universally leukoreduce blood components were implemented before the risk assessment was completed [49,59,62]. Among the majority who felt the risk assessment did not play a significant role in the decision-making process, the most common reason given was that the risk assessment involved too many uncertainties about which unreliable assumptions were made.

### Consideration of Leukoreduction as a Risk Management Strategy in the US

TSEAC also held its first meeting devoted to the transfusion transmission risk of vCJD in October 1997 [45]. The TSEAC meeting, however, followed a rather different format. Aguzzi's study was not presented. Instead, the focus in terms of laboratory data was on the preliminary results of a study conducted in the US by Paul Brown and Robert Rohwer. Brown and Rohwer had examined the distribution of prion infectivity in different blood components and plasma fractions [63]. Like Aguzzi's research results when they were first presented to SEAC, the results of their study were unpublished at the time. Also like Aguzzi's study, their study had come to the attention of regulators through social and professional connections.

Brown and Rohwer conducted their experiments in mice infected with Gerstmann-Straussler syndrome, pooling the blood from multiple animals and then separating and fractionating it in a scaled-down version of the process used by blood banks to process human blood [63]. They reported that while infectivity was present in buffy coat, it was also detectable in other blood components, such as plasma. In fact, it appeared that less than half of the total infectivity in blood was contained in the buffy coat. They admitted that this result came as a surprise to them [45].

While Brown and Rohwer's results were first presented to TSEAC in October 1997 [45], Aguzzi's results would not be presented to the committee until December 1998 [19]. By that time, many of the committee members were already aware of Aguzzi's results because they had been published [57]. At this meeting, Aguzzi interpreted the results more cautiously than he had in his publication, suggesting that while B-lymphocytes seemed to be required for infection, they might not carry prions [19]. At the same meeting, Rohwer again presented his finding that less than half of the infectivity in blood appeared to be associated with lymphocytes [19]. After much discussion, TSEAC recommended that deferrals be instituted for donors who spent a significant amount of time in the UK in order to decrease vCJD transfusion risk [19]. Leukoreduction, however, was not even suggested as a management strategy.

In June 2000, the FDA asked TSEAC to specifically consider the question of whether leukoreduction could be expected to reduce the risk of transmitting vCJD [44]. BPAC had recommended leukoreduction in 1998 for unrelated beneficial purposes: reducing non-hemolytic transfusion reactions and preventing the transmission of cytomegalovirus. Despite BPAC's recommendations, practical considerations, such as cost, had so far prevented the FDA from implementing regulations requiring universal leukoreduction. At this meeting, FDA asked TSEAC to exclude these benefits from its consideration and focus solely on vCJD.

Rohwer once again presented the results of the prion infectivity distribution experiment, indicating that less than half of the infectivity in blood was associated with the buffy coat [44]. While a member of Aguzzi's lab contributed to the meeting, he focused on the results of more recent experiments implicating the follicular dendritic cell, a long-lived component of the immune system that resides in the spleen and lymph nodes, as a potential site of prion replication [44]. He also presented the results of an experiment in which reconstituting the B-lymphocytes of PrP-expressing mice from PrP knock-out mice could re-establish susceptibility to prion infection [64]. He interpreted these results as indicating that B-lymphocytes do not carry infectivity themselves, but are required for the functioning of other components that do [44]. As cells that depend on signaling from B-lymphocytes for their maturation, follicular dendritic cells were an ideal candidate.

Results of parallel experiments in a different laboratory affirmed that PrP expression was not required by B-lymphocytes for infection with prion disease, suggesting that B-lymphocytes were unlikely to be the carrier of infectivity [65]. While not presented or discussed at the TSEAC meeting, at least three members of TSEAC were aware of these results at the time of the meeting.

When it came time for TSEAC to vote on whether leukoreduction could be expected to decrease significantly the infectivity theoretically present in the blood of persons with vCJD, the measure lost 13 to two [44]. Even the two members of the committee who voted in the affirmative both qualified their votes by stating that there was “insufficient information” at present to be assured of this conclusion.

More than three-quarters of the members of TSEAC and all but one of the US agency employees interviewed mentioned Brown and Rohwer's study when discussing the rationale for the decision not to require leukoreduction for the purpose of reducing vCJD transmission. While they were aware of Aguzzi's 1997 results, more than half of the members of TSEAC indicated that they were unsure of how to interpret them in light of further research. Four of the government employees interviewed indicated that Aguzzi's results had lost their allure by the point in time that TSEAC considered leukoreduction. One government employee specifically pointed out that the results of Brown and Rohwer's experiment negated the initial interpretation of Aguzzi's experiment that B-cells were the carrier of prions.

### Subsequent Events

In December 2003, the first probable transmission of vCJD through blood transfusion was announced [67]. The victim developed symptoms of vCJD 6.5 years after receiving a blood transfusion at the age of 62 [6]. One of the units of red cells he received had been donated by a 24-year-old man who later developed vCJD. Statisticians calculated the probability of recording a case of vCJD in this recipient in the absence of transfusion transmission to be from one in 15,000 to one in 30,000 [6]. In response to this case, the UK government announced a new policy excluding those who received blood transfusions after 1980 from donating blood [67].

Then, in August 2004, researchers published another worrisome case. They identified the disease-causing form of the prion protein (PrPres) in the spleen of a recipient of blood from a vCJD donor [7]. In this case, however, the patient had died of causes unrelated to vCJD. In fact, no PrPres had been identified in the patient's brain and there were no signs of neurological damage. Researchers questioned whether he would have gone on to develop the disease had he lived long enough [7]. In addition, this patient was of a different genotype than all other patients who had contracted vCJD. He was heterozygous for methionine/valine at codon 129 of the prion protein gene [7]. This raised concern about whether a larger subsection of the UK population might be at risk for contracting the disease than was previously thought.

Even though the majority of research now indicates that lymphocytes are unlikely to be the carriers of prion infectivity in blood, the UK has not rescinded its policy of leukoreduction. This is reconcilable with the fact that leukoreduction has only increased in popularity around the world since the UK made its decision, largely due to the reputation of leukoreduction as a process capable of reducing problematic transfusion reactions [68–70]. The UK continues its policy of universal leukoreduction, and many other European countries and Canada have instituted the process as well [71–73]. In the US, multiple hospitals and blood banking services have implemented their own leukoreduction policies despite the fact that it is not an FDA requirement [74].

## Discussion

Qualitative case studies, such as the one described in this article, provide an in-depth exploration of phenomena bounded by space and time [75]. As such, the results of a single case study are not expected to hold true universally. They may be thought of as generalizable, however, in that they attune us to certain possibilities and provide a framework for analyzing similar phenomena [76]. The term “theoretical insight” will be used in place of “conclusion” in the following discussion in order to delineate this difference. While such theoretical insights should not be expected to apply in all cases, they are useful in that they provide a mechanism for anticipating possible future issues in the management of emerging infectious disease.

In the past three decades, considerable efforts have been made at the international level to enhance the science-based quality of risk management. These efforts have been driven by the desire to harmonize regulations and generate solidarity among nations, especially when issues of trade are involved [77]. Such harmonization is thought to be important to the smooth functioning of a free market. In this vein, the 1995 Sanitary and Phytosanitary Act of the World Trade Organization called for all nations to base their food and consumer product safety regulations on science [78].

While science is indeed an ingrained component of disease management policy in many countries, science is not the only factor that influences disease management policy. Recent experience with other infectious diseases can have a substantial impact on the direction and focus of disease management policy. For example, the propensity to issue recalls of blood and blood products in Western countries for fear of transfusion transmission risk is influenced by the experience with HIV/AIDS [79,80]. Past experience is invoked both by affected communities as well as relevant regulators and scientists. Such was the case with vCJD, where recent experience with transfusion transmission of HIV generated a context of heightened concern about other potential risks to the blood supply, leading to both recalls and the institution of policy changes. In contrast, recent experience with infectious disease had the opposite effect when HIV was first recognized. In this case, prior experience with swine flu [41] and hepatitis B [81] led to the inappropriate minimization of blood transfusion concerns. This leads us to our first theoretical insight: because the perception and management of disease may be shaped by previous experience, especially catastrophic experience, there is always the possibility for over-emphasis of certain potential routes of transmission and under-emphasis of others.

In the above case study, UK government employees and scientific advisors were under substantially greater time pressure and approached issues pertaining to vCJD with more urgency than their US counterparts. The public in the UK felt that they had been duped by their government about the threat of BSE, leading to calls for an inquiry into the government's management of the disease. In addition, the UK government's recall of blood products for possible vCJD contamination increased the sense of urgency in that country. This sense of urgency led the UK government to implement policies within a relatively short time frame. Because BSE and vCJD had not yet reached the US, US officials and scientific advisors experienced less time pressure in considering possible policy solutions, allowing for greater reflection on the data and for the development of further scientific results before making their decisions. Thus, the US did not impose its first set of deferrals until November 1999, more than a year after the UK had implemented both its plasma importation and universal leukoreduction policies. Our second theoretical insight follows from this observation: local specificities may influence the temporality and direction of disease management.

In addition, and contrary to published opinion [77], the growing reliance on science in determining appropriate risk management strategies does not ensure policy agreement. As exhibited in this case study, the differential uptake and interpretation of scientific studies can result in contradictory policy decisions. This differential uptake may result from the existence of alternative social and professional networks, which promote or allow access to certain scientific studies, especially unpublished ones, at different points in time. In other words, what Choi refers to as “knowledge exchange,” or the transfer of information between science and policy [82], occurs along networks that are social in character. This is not a new insight, but one that has been recognized by scholars in the discipline of science and technology studies for some time [83–85]. Social networks influence decisions about who is invited to present research at advisory committee meetings, how many times they are invited to present, and the manner in which committee members refer to certain research results during discussions.

The resulting differences in exposure can have important consequences for the interpretation of results and subsequent decision making. Such was the case with vCJD, where an alternate sequence of exposure to scientific studies and a relative imbalance in the prominence of different results due to existing social networks led to contradictory scientific interpretations of the utility of leukoreduction as a risk management strategy. This leads to our third theoretical insight: basing risk management decisions on science will not necessarily lead to homogeneous policies amongst nations because the exposure to and interpretation of scientific results depends on existing social and professional networks.

### Qualitative Research as a Technique for Investigating Complex Social Phenomena in Medicine and Health Policy

While quantitative methods are ideal for testing hypotheses regarding simple causal relationships, there are certain research questions, such as the questions asked in this study, that are not readily amenable to quantitative forms of investigation. Other unrelated but important questions in health care and health policy today, such as how physicians decide whether to classify an event as a medical error, provide similar challenges. Because these questions revolve around the organization and culture of institutions and the people who occupy them, they require the application of in-depth, broad-based, qualitative methods.

Qualitative methods are sometimes viewed with suspicion by clinicians who are more familiar with quantitative methods. It should be remembered, however, that no form of research is free from the influence of bias and personal assumptions. As Ioannidis points out, quantitative studies are habitually plagued by such issues, and they cannot be eliminated simply by conducting more studies or involving independent research teams [86]. “A major problem,” he notes, “is that it is impossible to know with 100% certainty what the truth is in any research question.”

Popay has suggested that the primary marker of high-quality qualitative research is its ability to produce a provisional understanding of the basis of behavior and action [87]. As such, good evidence in qualitative research is often held to depend on a thorough investigation of subjective meaning. In the above study, this was achieved through a lengthy period of immersion (three years) in the field of prion research and prion policy production. This immersion involved multiple discussions with members of advisory committees, agency employees, and scientists as well as participation and observation at multiple scientific meetings, advisory committee meetings, and other forums for the discussion of issues related to the topic of study.

While quantitative research aims for reliability through the use of standardized mechanisms of data collection, such as surveys, these tools are antithetical to qualitative research [88]. Because most qualitative research is inductive, it is important not to impose ready-made categories on the process of data collection, which is precisely what the application of a survey or a standardized interview format does. Thus, while a transparent description of methodology is key to ensuring reliability in qualitative research [88], qualitative methods will never achieve the level of reliability inherent in quantitative methods due to their interpretive nature. This should not, however, negate the utility of qualitative research. The complexity of interpreting and evaluating qualitative research is a by-product of the same characteristics that make qualitative methods suitable for the investigation of otherwise impenetrable problems in medicine and health policy. In other words, this shortcoming can be viewed as an opportunity.

The three theoretical insights outlined in this paper: that the perception and management of disease may be shaped by previous experience, that local specificities influence the temporality of decision making, and that the exposure to and interpretation of scientific results may depend on existing social and professional networks within a given nation, provide a frame-work for analyzing differences in disease management policy across nations. They also provide a mechanism for anticipating future international management issues concerning emerging threats to human health, such as SARS, H5N1 influenza, and even bioterrorism. The above analysis indicates that improved dissemination and uptake of science-based criteria is not enough to ensure solidarity. Thus, it is important to have other mechanisms in place for enhancing cooperation when cooperation is desired.

## Abbreviations

BSE: bovine spongiform encephalopathy
sCJD: sporadic Creutzfeldt-Jacob disease
vCJD: variant Creutzfeldt-Jakob disease
